# A Systematic Review Comparing the Play Profiles of Children with Special Health Care Needs with Typically Developing Children

**DOI:** 10.1155/2020/9582795

**Published:** 2020-11-19

**Authors:** Nyaradzai Munambah, Reinie Cordier, Renée Speyer, Sivuyisiwe Toto, Elelwani L. Ramugondo

**Affiliations:** ^1^Department of Rehabilitation, University of Zimbabwe, Harare, Zimbabwe; ^2^Department of Social Work, Education and Community Wellbeing, Northumbria University, Newcastle upon Tyne, UK; ^3^School of Occupational Therapy, Social Work and Speech Pathology, Faculty of Health Sciences, Curtin University, Perth, Australia; ^4^Department of Otorhinolaryngology and Head and Neck Surgery, Leiden University Medical Centre, Leiden, Netherlands; ^5^Department Special Needs Education, University of Oslo, Oslo, Norway; ^6^School of Public Health and Family Medicine, Faculty of Health Sciences, University of Cape Town, South Africa; ^7^Department of Rehabilitation Sciences, Faculty of Health Sciences, University of Cape Town, South Africa

## Abstract

**Introduction:**

Although play has been used as a means to meet therapeutic goals by health care practitioners for a long time, there is a need to continuously review its conceptualisation and use in everyday practice to promote evidence-based practice. This systematic review aimed to evaluate the evidence on how the play of children with Special Health Care Needs (SHCN) is similar or different to that of typically developing children.

**Methods:**

Guided by the preferred reporting items for systematic reviews and meta-analyses (PRISMA) statement, we conducted a comprehensive review across five electronic databases for all studies that compared how the play of children with SHCN was similar or different to that of typically developing children. Data were extracted from the included studies, and methodological quality was assessed.

**Results:**

Eighteen studies met eligibility criteria. All the studies in this review were at risk of bias due to the study design. There was great variation in sample sizes, ranging between five and 112 participants in the diagnostic groups and five and 546 participants in control groups (typically developing children). The included studies investigated different aspects of play, which made it difficult to synthesise. However, of the 18 studies reviewed, thirteen reported that children with SHCN engage in less play, compared with typically developing children.

**Conclusions:**

Evidence supports the assumption that children with SHCN are less playful and spend less time engaging in play compared with typically developing children. This systematic review reveals paucity of research on play for children with several common chronic conditions such as HIV/AIDS, cancer, and cardiovascular diseases. Future studies need to reduce risks of bias, including the use of appropriate sample sizes, and must provide detailed results after investigating play in children with SHCN.

## 1. Introduction

Generally, there is an increase in children with Special Health Care Needs (SHCN) ([1]); consequently, more focus has been on improving services to this population [2]. Some of the commonly identified health care conditions among children include ADHD, asthma, learning disability, and cerebral palsy ([3]). Children with special healthcare needs have been defined as “children who have or are at risk for a chronic physical, developmental, behavioural, or emotional condition, and who also require health and related services of a type or amount beyond that required by children generally” ([4], 138). Although there are various ways of defining children with SHCN, the above definition was selected for the current systematic review, because it is more inclusive and captures the potential service needs of these children as compared to other definitions used in literature to capture the different types of health conditions and related participation limitations [5].

Despite the great variability in terms of the types and severity of special healthcare needs, children with SHCN experience multiple health conditions, poorer reported health status, and reduced physical activity among other challenges [5]. Children with SHCN are at risk of mental and behavioural problems, frequent readmissions to hospital, absence from school, and limited capacity to engage in play (Van [6]). Thus, children with SHCN are likely to experience challenges in their development, and some of the common problems they experience include difficulty in learning and paying attention (L.S. [3]). As such, children with special healthcare needs are at greater risk than their healthy peers of experiencing adverse learning and developmental outcomes both in the short and longer terms. All these adverse outcomes point towards the need for early intervention to minimise the impact of the condition. For children, play is commonly the medium for intervention delivery and an important therapeutic outcome in and of itself [7].

Play-based interventions are part of the several approaches that have been utilised by healthcare professionals to address deficits experienced by children with SHCN [8]. Play has been used in therapy for a long time, as either a means to address deficits or promoted as an end goal. Through play, children learn survival skills and develop resilience to deal with life events and all skills that are essential for transitioning into adulthood [9]. Play is also an important resource for learning and developing critical motor, cognitive, and socioemotional skills [7]. Furthermore, play provides a natural context to explore behavioural and social difficulties, in addition to addressing interactional problems that affect children [10].

Even though play has been used as both a medium for intervention and a therapeutic agent for a very long time [7], as a multifaceted phenomenon, the construct remains elusive to define, given the dynamic and constantly evolving nature of the construct. A study by Ramugondo [11] revealed how play progressed and changed from one generation to the next. The study highlighted the importance of context in shaping play. The context in terms of who plays with the child, availability of playthings ([12]), and play spaces, as well as culture, influences the play of children. As children grow and develop more complex forms of play, they tend to develop certain gender preferences in the selection of toys as facilitated through their context [13]. Play is also shaped by culture ([14]); hence, the types and forms of play may vary from one culture to the next. A study by Berinstein and Magalhaes [15] reported that “…play of children in Tanzania was different from the western perspective of play”. As a result, the complexity, diversity, and constantly changing nature of play make measuring play ability difficult for educators, clinicians, and researchers [16].

The conceptualisation of play has been drawn from many disciplines to describe play at different stages, with implications for both assessment and practice [16]. Although there is some disagreement about the exact characteristics that comprise play, play is commonly defined by the characteristics that separate it from nonplay [17]. For the purposes of this systematic review, play is defined as “...a transaction between the individual and the environment that is intrinsically motivated, internally controlled, and free of many of the constraints of objective reality and skills related to framing (giving and responding to cues)” ([18], 227). Rather than viewing play in general terms, this review also includes studies that have focused on playfulness. Playfulness is a key aspect of play to explore in children with impairments, as it focuses on the quality of play and the adaptability and coping mechanisms of a child, regardless of ability. Playfulness is defined as a child's disposition to play, which remains constant over time and relates to a child's ability to cope in later life [19]. The construct of playfulness is characterised by four elements which are intrinsic motivation, internal control, freedom to suspend reality, and framing [19]. Although play refers to the “doing” of play, playfulness points to the very “being” of play [17]. The terms play, playfulness, play profiles, and play behaviours have been used to denote play or various forms of play in literature. For the purpose of this study, the term play profiles will be used henceforth where appropriate.

Despite play being spontaneous, children with SHCN are likely to experience difficulties in engaging and participating in play [7]. Restrictions to being able to play well with others can lead to children developing negative self-efficacy and becoming socially isolated [17, 20] which, in turn, may result in further psychological sequelae later in life. Play is the window through which a child's development can be viewed, and by exploring their play profiles, we can develop a clearer picture of how other areas of development may be affected. An understanding of the play profiles of children with special healthcare needs would be important in developing appropriate and evidence-based interventions for this population. However, there is paucity of high-level evidence on the impact of SHCN on children's play [21, 22]. High-level evidence is needed to inform therapy needs and making evidence-informed choices about the quality of interventions offered to children with special healthcare needs. Systematic reviews provide high-level evidence by synthesising information from all accessible studies [23], which can be used to guide practice and support development of appropriate strategies to improve play in children with SHCN. This systematic review aims to examine how the play profiles of children with SHCN, which is similar or different to that of typically developing children. The systematic review addressed the following research questions:

1. Is the overall play profile of children with SHCN similar or different to that of typically developing children?
2. Is the play duration of children with SHCN similar or different to that of typically developing children?
3. Are the types and/or forms of play of children with SHCN similar or different to that of typically developing children?
4. Is the play behaviours of children with SHCN similar or different to that of typically developing children?

## 2. Materials and Methods

PRISMA was used to guide the methodology and transparent reporting of this systematic review [24]. The PRISMA checklist describes aspects of research that are deemed essential for the transparent reporting of systematic reviews. This systematic review is registered with PROSPERO International Prospective Register of Systematic Reviews (ID 2017: CRD42017072269). The systematic review addresses the research question: how is the play of children with SHCN similar or different to that of typically developing children?

### 2.1. Selection Criteria

The study population included children aged 11 years and younger with (1) specified SHCN such as HIV/AIDS, chronic respiratory conditions, cancer, and physical disabilities, such as cerebral palsy and spina bifida; and (2) behavioural and/or emotional disorders which are defined as disruptive impulsive conduct disorders and anxiety disorders according to the DSM 5. However, autism spectrum disorders were excluded from this study as a systematic review on play-based interventions for children with ASD was conducted by Kent et al. [16], and a review of play interventions for children with autism at school was carried out by Kossyvaki and Papoudi [25].

The inclusion criteria for the studies were as follows:

- The studies could be of any design provided play was compared between two groups of children
- At least one of the comparison groups should be children diagnosed with specified special healthcare needs, compared against typically developing children
- At least one of the outcome measures should be play; and
- Articles were published in English and in peer-reviewed journals

The exclusion criteria were as follows:

- Studies that focused on caregivers and/or siblings of participants who meet eligibility criteria
- Data from presentations, conference proceedings, dissertations, and thesis
- Studies that reported on a play-based intervention but did not measure play as an outcome
- Studies that investigated play in which video games and robotics are used. Play involving video games and robotics is structured and may not provide similar opportunities for social interaction, freedom to suspend reality and framing, and restricts the children to being creative outside the confines of the game/robotics. Moreover, as we were interested in understanding the play profiles of children with SHCN, the play context (naturalistic) needed to be consistent to allow for comparison across different population groups
- Studies that used a psychoanalytical approach to play or so-called “play-therapy” approaches. According to [26], play therapy is a way of helping troubled children copes with their distress, using play as the medium of communication between the child and the therapist. Thus, play therapy is not an intervention aimed at addressing play deficits per se but is mainly focused on addressing emotional distress

### 2.2. Information Sources and Search

A systematic literature search was conducted across the following five databases: CINAHL, Embase, ERIC, PsycINFO, and PubMed. The searches were done between September 22 and December 10, 2018. Additionally, reference lists of included studies were searched by hand to ensure that all appropriate articles were included in the review. All search strategies per database are presented in Table 1.

### 2.3. Data Extraction (Selection and Coding)

Through consultations with an expert panel experienced in play research and/or measurement development, a data extraction form was developed based on the review question. Variables sought from the checklist included study setting, study population, participant demographics, diagnosis of participants, play/playfulness outcome measures used, and the methodological quality of the included studies. Data on the methodological quality of the studies were extracted and scored independently by reviewers who were not authors of any of the studies included in the review. Authors of the included studies were contacted if additional information was needed to answer questions with regard to eligibility.

### 2.4. Risk of Bias (Quality) Assessments

Assessment of methodological quality of the studies included was done using the Kmet Appraisal checklist, also referred to as “QualSyst” [27]. The Kmet Appraisal checklist was used because it is suitable for assessing quality across a broad range of study designs. The checklist uses ordinal ratings to score reported information (i.e., yes = 2, partial = 1, or no = 0). A score of “not applicable” reduces the possible total Kmet sum of scores, which can be calculated to a percentage score. A score of >80% is considered strong quality, 60-79% good quality, 50-59% adequate quality, and less than 50% poor quality. Interrater reliability for abstract selection and Kmet ratings was established by two independent assessors based on weighted kappa calculations. There was no evident bias in scoring study quality and extractor bias of the reviewers conducting this systematic review.

### 2.5. Data Analysis

A narrative-synthesis was selected to obtain meaningful interpretation of the findings of the included studies and to describe how play is similar or different in children with SHCN, children with behavioural and/or emotional disorders, and children with physical disabilities when compared with children who are typically developing.

## 3. Results

### 3.1. Study Selection

**Figure 1:**
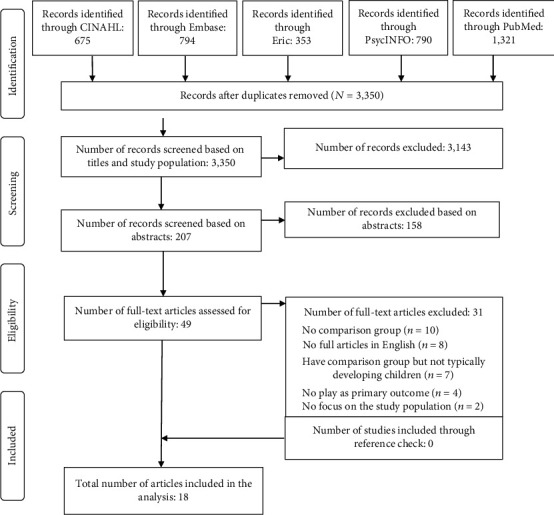
PRISMA flow diagram.

A total of 3,933 abstracts were retrieved.  Figure 1 presents a flow diagram of the abstract reviewing process. The number of records retrieved from each database was CINAHL = 675, Embase = 794, ERIC = 353, PsycINFO = 790, and PubMed = 1,321. No study was identified through hand search of the reference lists of identified articles. A total of 583 duplicates were removed across the databases, resulting in 3,350 independent studies. Two independent researchers screened the 3,350 records for inclusion by title and abstracts; 207 full-text articles were assessed for eligibility. Disagreements between the two reviewers were resolved by consensus, and a third reviewer was consulted if agreement could not be reached between the first and second reviewers. In total, 49 studies were identified from full articles. A list of studies published in peer-reviewed journals that were excluded and reasons for their exclusion are presented in Supplementary Table 1 provided. Based on the inclusion criteria, 18 studies were selected for inclusion in this review.

### 3.2. Methodological Quality

Eighteen studies published between 1990 and 2018 that compared play in children with SHCN against typically developing children were identified. Of these selected studies, four studies used a quasi-experimental study design [20, 28–30], and thirteen were cross-sectional studies ([17, 31–40]; S [10, 41]). A study by Wilkes-Gillan et al. [42] used a multiple case study design. All the studies included in this systematic review reported adherence to ethical principles to a reasonable extent. Ethical principles reported included obtaining consent from participants, maintaining confidentiality, and promoting the safety of children throughout the data collection process. Based on the National Health and Medical Research Council (NHMRC) Hierarchy of Evidence (National [43]), five of the studies reviewed were classified as level III, and thirteen were classified as level IV evidence. According to the NHMRC Hierarchy of Evidence, level I studies are systematic reviews of randomised controlled trials (RCTs), level II studies are well-designed RCTs, and level III studies are, for example, quasiexperimental designs without random allocation. The overall methodological quality ranged from good to strong, with five studies ranked as good and thirteen as strong according to the Kmet ratings.

### 3.3. Risk of Bias

All the studies in this review were at risk of bias due to study design. Twelve of the 18 studies used a cross-sectional study design, four used a quasi-experimental design, one used a longitudinal design, and another one used a multiple case study design. Of the 18 studies, five had small sample sizes of <30 participants, which limits the generalisability of the findings [20, 34, 36, 37, 42]. Allocation concealment was not possible, because all the studies reviewed compared play/playfulness of children diagnosed with a specific condition against typically developing children. Thus, on recruitment, it was possible for the participants to know which group they belonged to. Also, as none of the studies had an RCT design, participants were not randomly assigned to different groups. Generally, all studies were at risk of confounding bias.

### 3.4. Participants

The 18 studies included in this review had a total of 1,608 participants aged between 8 months and 13 years, with 67.5% being males. Despite adopting a broad search strategy to capture all chronic illness, only studies with the following diagnosis were found: 184 had ADHD, 125 had general developmental delays, 99 had developmental coordination disorder, 79 had cerebral palsy, 31 had cognitive and speech disorders, 15 had prenatal alcohol exposure, and 14 had Down syndrome.

### 3.5. Playmates

All studies reported that participants were observed either playing alone, with another child or with an adult; a summary is provided in Supplementary Tables 2 and 3. Five studies reported to have observed children from the diagnostic groups playing with a playmate and, in most cases, the playmate was a child who was familiar to the child ([20, 39, 42]; S [10, 41]). Four of five studies that reported on playmates included siblings as the playmates. The proportion of playmates that were siblings was 60% Cordier et al. [10], 80% Wilkes-Gillan et al. [42], and 62% Barnes et al. [20], and Venkatesan and Ravindran [41] had 100%. In some studies, children played in the presence of adults who were either the parent [29, 30, 37] or a caregiver [35]. Five studies observed children playing with their peers in the school setting (either classroom or playgrounds) [17, 31, 34, 36, 40]. Four studies reported that participants were observed playing alone [28, 32, 33, 38].

### 3.6. Study Settings

Participants were observed playing either at home, school, or at a clinic. Seven of the 18 studies reported that participants were observed at school. Of these seven studies, three studies were conducted in the classroom [28, 31, 34], two studies were conducted in the playgrounds [17, 39], and two studies were conducted both in the classroom and in the playground [36, 40]. Two studies observed typically developing children in a school environment and the diagnostic group in the clinic [10, 32]. Two studies were carried out in the home setting [30, 35], while two studies [20, 38] observed the children in both the home and the clinic environment. A study by Barnes et al. [20] used a playroom at the clinic to carry out their observations. Two of the 18 studies observed the children playing in a custom-designed playroom [29, 37]. Only two studies did not report the settings where the child was observed playing [33, 41].

### 3.7. Observation Time

The observation time varied from 10 seconds to 60 minutes per session. Seven of the 18 studies observed each participant for between 15 and 20 minutes [10, 17, 20, 30, 35, 39]. Observation time was not reported in four studies ([29, 32, 33]; S [41]).

### 3.8. Play Assessment Tools

Although all the studies reviewed involved observing children playing, different assessment tools were used. The Test of Playfulness [19] was used in six of the 18 studies reviewed [10, 17, 20, 30, 35]. Other assessment tools used include the revised Knox Preschool Play Scale (KPPSr; [44]) which was used by Angelin et al. [32], the Play Scale-Brief Rating (APS-BR) [45] used by Hsieh [28], and the Play Activity Checklist for children with Mental Retardation (PACK-MR; [46]) used by Venkatesan and Ravindran [41].

One study by Cairney et al. [33] used a participation questionnaire to collect data on active play. Eight studies used various coding approaches to record and analyse play among children [29, 31, 34, 36–40]. For example, the study by Skinner et al. [39] used Parten's social play categories [47], and Hestenes and Carroll [36] used a scan-sampling technique developed by Nabors [48]. A study by Smyth and Anderson [40] used the Psion Workabout computer using a detailed coding scheme developed in the Observer software system for coding [49]. The coding systems and behaviours were operationally defined differently across all the studies that involved behavioural coding, indicating a lack of evidence of validity. However, interrater reliability of observers was established for some of the studies ranging between 80 and 100% agreement. Most studies in this review were at risk of observation bias due to a lack of blinding of the researchers for outcomes.

### 3.9. Play of Children with SHCN

The studies reviewed examined different aspects of play, which makes it difficult to synthesise the findings. However, for the purposes of comparing play patterns, findings were extracted and grouped into four themes: overall play, play duration, types of play, and play behaviours (see Table 2). Of the 18 studies included in this systematic review, 15 studies reported on the differences in play patterns between the various diagnostic groups and typically developing children in the control groups. Eight of the fifteen studies found that typically developing children were overall more playful than children with ADHD [10, 31], CP and/or DCD [30, 32], and developmental delays and other disabilities [17, 35, 38, 39]. Five studies reported on the duration the child remained engaged in play and found that typically developing children spent more time engaged in play compared with children with ADHD [31], physical conditions [33, 40], and developmental delays and other disabilities [36, 37].

Types of play were reported differently across all studies, and most were reported as frequency of occurrence of that type of play. Three of the five studies involving children with ADHD reported that children with ADHD engage less in social play as compared with typically developing children [10, 20, 42]. One study involving children with physical disabilities [40] and another involving developmental delays [17] investigated children's social play. Both studies reported that target children engaged in less social play compared with typically developing children. Compared to typically developing children, children with physical conditions engaged in less gross motor play [32], less pretend play [28], and less in play involving games [40]. Conversely, children with developmental delays and other disabilities engaged more in solitary or parallel play [34, 36] as compared with typically developing children.

In terms of play behaviours, only one of the five studies on ADHD [31], three of the five studies on physical conditions [28, 32, 40], and four of the eight studies involving developmental disabilities [34, 36–38] reported on play behaviours. Compared to typically developing children, children in the diagnostic groups engaged in more interrupted play [31], presented with more onlooker behaviours [36, 37], and displayed less complex play [34, 38, 40].

## 4. Discussion

This is the first systematic review to examine how the play profile of children with SHCN is similar or different to that of typically developing children. Although play can be used as a medium to assess children's development, the focus of this review was limited to studies that used play as both an outcome and a medium for intervention for children with SHCN within naturalistic contexts and compared this to typically developing children. Play is a multidimensional and complex construct which is difficult to define [16]. Thus, the process of focusing the search enabled researchers to compare and contrast the play profiles of children with SHCN to that of typically developing children. A total of eighteen studies met the inclusion criteria. This was surprising, given the high number of studies that investigated play in children and more so the importance of play in everyday doing of children and that play has been used in therapy for a long time [9]. However, the number of studies included in this systematic review, is in keeping with number of studies included in other recently published systematic reviews in this field. For example, a systematic review by Kent et al. [16] included 19 studies, and a systematic review by Watts et al. [50] on play and sensory processing included 8 studies.

Although the search strategy was broad to capture all types of conditions that require SHCN in children, the impact of SHCN on the play of children has only been investigated for the following conditions: ADHD, physical conditions (cerebral palsy and developmental coordination disorder), and developmental delays and other disabilities such as cognitive and speech disorders, Down syndrome, and prenatal alcohol disorders. By defining children with SHCN as children who have or are at risk of physical, developmental, behavioural, and emotional difficulties that require more health or related services as compared to typically developing children [4], this review reveals a paucity of research in play for children with several common conditions such as asthma, cystic fibrosis, HIV/AIDS, cancer, and cardiovascular diseases. Research on play in children with SHCN is very important as these conditions pose a risk of limitations in performance of daily activities and play. This is particularly pertinent in less economically developed countries where research points to an increase in the prevalence of conditions such HIV/AIDS and behaviour and/or learning problems.

The main finding of this review is that sixteen of the eighteen studies reported that children diagnosed with SHCN are generally less playful when compared with typically developing children. This finding supports the notion that conditions requiring special health care in children influence a child's developmental trajectory, including play [2]. Due to illness, children with SHCN are likely to experience more restrictions, fewer opportunities, less access, and less range of ability for play compared to other children [9]. Thus, there is need of interventions aimed at promoting play in this population.

Studies reviewed reported play in a variety of ways, which makes it difficult to compare. However, the authors' synthesised the findings against the following four themes: overall playfulness, time engaging in play, types of play, and play behaviours. In terms of types of play, five of the 18 studies reported that children with special health care needs engaged in less social play as compared with typically developing children. Similarly, in a systematic review by Kossyvaki and Papoudi [25] on play intervention for children with autism in schools, social play was investigated in six of the 14 studies. This could be because most studies used the Test of Playfulness to report on social play. Using standardised measurements allows for uniform reporting, allowing comparison and generalisability to different contexts [35]. This review points to the limited number of measures used in the evaluation of play with eight out of 16 studies using standardised assessments while the other nine studies used a coding system. Most of the coding systems used were specifically designed for that particular study without psychometric details being reported on the validation of the coding system. Using a coding system in the absence of psychometric details has a higher risk of bias as compared with validated tools with sound psychometrics.

Participants were observed playing in natural environments where they normally play, and these include the home, school, or at a clinic. The setting was important as research on play in children is best done through observations of children in their naturalistic environment [9]. Children tend to play more in familiar environments [35] and with playthings such as toys (Srinivasan [12]). The environment has a potential to either stimulate or inhibit play as children tend to play in safe and comfortable environments [10]. Findings from this review point to a need to observe the child playing with other children ([7, 18]); however, there is a need for further research on the effects of the presence of a playmate in play.

Although this systematic review gives direction on what research has been done, and points out gaps in areas that need more research focus, most of the studies used small sample sizes making it difficult to generalise the findings.

### 4.1. Limitations of the Study

There are a number of potential limitations of this study. Due to the heterogeneous nature of the studies and outcome measures, it was not possible to conduct a meta-analysis on the similarities and differences in the play of children with SHCN compared with typically developing children. Inclusion was limited to studies published in English only. Three of the authors in this review were also coauthors of three studies included. However, bias was minimised by asking two independent reviewers to screen and select studies to be included in this study. Most studies included in the review were at high risk of bias due to the study design, lack of randomisation, and inadequate blinding.

## 5. Conclusion and Implications for Future Research

Although play is viewed as a childhood occupation that is spontaneous [7], play in children with SHCN can be limited in frequency, quality, and limited in repertoire. This systematic review reveals a paucity of research on play for children with several common chronic conditions such as HIV/AIDS, cancer, and cardiovascular diseases. There is a need for more research on the play of children with SHCN; especially in low developed countries where the population of children with SHCN is increasing. Findings from studies included in this review point to children with SHCN being less playful when compared to typically developing children. Furthermore, children with SHCN spent less time engaged in play compared to typically developing children. Future research needs to use more rigorous research designs and standardised play outcome measures, as this will allow for comparison and generalisability of findings to other contexts.

## Figures and Tables

**Table 1:** Search strategies per literature database.

Database	Subject headings	Number of records
CINAHL	((MH “Intellectual Disability”) OR (MH “Learning Disorders”) OR (MH “Motor Skills Disorders”) OR (MH “Movement Disorders”) OR (MH “Apraxia, Developmental”) OR (MH “Brain Injuries”) OR (MH “Cerebral Palsy”) OR (MH “Hydrocephalus”) OR (MH “Fragile X Syndrome”) OR (MH “Down Syndrome”) OR (MH “Attention Deficit Hyperactivity Disorder”) OR (MH “Fetal Alcohol Syndrome”) OR (MH “Social Behavior Disorders”) OR (MH “Affective Disorders”) OR (MH “Child Behavior Disorders”) OR (MH “Developmental Disabilities”) OR (MH “Learning Disorders”)) AND ((MH “Play Therapy”) OR (MH “Play and Playthings”) OR (MH “Play Therapy (Iowa NIC)”) OR (MH “Play Participation (Iowa NOC)”) OR (MH “Role Playing”))	675
Embase	(intellectual impairment/OR mental deficiency/OR motor dysfunction/OR developmental coordination disorder/OR tic/OR apraxia/OR traumatic brain injury/OR cerebral palsy/OR hydrocephalus/OR fragile X syndrome/OR Down syndrome/OR attention deficit disorder/OR fetal alcohol syndrome/OR emotional disorder/OR conduct disorder/OR oppositional defiant disorder/OR behavior disorder/OR developmental disorder/OR behavior disorder/OR learning disorder/OR hyperkinesia/OR antisocial personality disorder/) AND (play/OR play therapy/)	794
ERIC	(MAINSUBJECT.EXACT(“Intellectual Disability”) OR MAINSUBJECT.EXACT(“Severe Intellectual Disability”) OR MAINSUBJECT.EXACT(“Moderate Intellectual Disability”) OR MAINSUBJECT.EXACT(“Mild Intellectual Disability”) OR MAINSUBJECT.EXACT(“Developmental Disabilities”) OR MAINSUBJECT.EXACT(“Attention Deficit Hyperactivity Disorder”) OR MAINSUBJECT.EXACT(“Attention Deficit Disorders”) OR MAINSUBJECT.EXACT(“Neurological Impairments”) OR MAINSUBJECT.EXACT(“Cerebral Palsy”) OR MAINSUBJECT.EXACT(“Down Syndrome”) OR MAINSUBJECT.EXACT(“Fetal Alcohol Syndrome”) OR MAINSUBJECT.EXACT(“Emotional Disturbances”) OR MAINSUBJECT.EXACT(“Behavior Disorders”) OR MAINSUBJECT.EXACT(“Developmental Disabilities”) OR MAINSUBJECT.EXACT(“Minimal Brain Dysfunction”) OR MAINSUBJECT.EXACT(“Learning Problems”) OR MAINSUBJECT.EXACT(“Learning Disabilities”) OR MAINSUBJECT.EXACT(“Hyperactivity”)) AND (MAINSUBJECT.EXACT(“Play”) OR MAINSUBJECT.EXACT(“Play Therapy”))	353
PsycINFO	(Intellectual Development Disorder/OR “Intellectual Development Disorder (Attitudes Toward)”/OR Learning Disabilities/OR Learning Disorders/OR Developmental Disabilities/OR Dyspraxia/OR Hyperkinesis/OR Movement Disorders/OR Cerebral Palsy/OR Traumatic Brain Injury/OR Hydrocephalus/OR Fragile X Syndrome/OR Down's Syndrome/OR Attention Deficit Disorder with Hyperactivity/OR Attention Deficit Disorder/OR Prenatal Exposure/OR Fetal Alcohol Syndrome/OR Affective Disorders/OR Emotional Disturbances/OR Conduct Disorder/OR Oppositional Defiant Disorder/OR Behavior Disorders/OR Developmental Disabilities/OR Learning Disabilities/OR Learning Disorders/) AND (Childhood Play Behavior/OR Pretend Play/OR Play Therapy/OR Role Playing/OR Playfulness/)	790
PubMed	(“Intellectual Disability”[Mesh] OR “Mental Retardation, X-Linked”[Mesh] OR “Learning Disorders”[Mesh] OR “Motor Disorders”[Mesh] OR “Motor Skills Disorders”[Mesh] OR “Stereotypic Movement Disorder”[Mesh] OR “Tic Disorders”[Mesh] OR “Apraxias”[Mesh] OR “Apraxia, Ideomotor”[Mesh] OR “Gait Apraxia”[Mesh] OR “Brain Injuries, Traumatic”[Mesh] OR “Cerebral Palsy”[Mesh] OR “Hydrocephalus”[Mesh] OR “Hydrocephalus, Normal Pressure”[Mesh] OR “Fragile X Syndrome”[Mesh] OR “Down Syndrome”[Mesh] OR “Attention Deficit Disorder with Hyperactivity”[Mesh] OR “Fetal Alcohol Spectrum Disorders”[Mesh] OR “Affective Symptoms”[Mesh] OR “Conduct Disorder”[Mesh] OR “Attention Deficit and Disruptive Behavior Disorders”[Mesh] OR “Social Behavior Disorders”[Mesh] OR “Child Behavior Disorders”[Mesh] OR “Developmental Disabilities”[Mesh] OR “Specific Learning Disorder”[Mesh] OR “Hyperkinesis”[Mesh]) AND (“Play Therapy”[Mesh] OR “Play and Playthings”[Mesh])	1321

**Table 2:** Nuances of play.

Theme	Play descriptors	Typically developing children	ADHD^a^ (5)	Physical (CP^b^ and DCD^c^ (5)	Developmental delay and other disabilities
Overall playfulness	More playful	▲	▼ (2)	▼ (2)	▼ (4)
Play duration	More time engaged in play	▲	▼ (1)	▼ (2)	▼ (2)
	Time playing alone	▼		▲ (1)	▲ (1)
Types/forms of play	Solitary or parallel play	▼			▲ (2)
	Constructive rather than functional play	▲	▼ (1)		▼ (2)
	Cooperative play	▲			▼ (2)
	Gross motor play	▲		▼ (1)	▼ (1)
	Sensory play	▼	▲ (1)		
	Pretend or dramatic play	▲		▼ (1)	▼ (2)
	Social play	▲	▼ (3)	▼ (1)	▼ (2)
	Playing games			▼ (1)	
Play behaviours	Emotional expression	▲		▼ (1)	
	Imagination	▲		▼ (1)	
	Onlooker behaviours	▼		▲ (1)	▲ (1)
	Play interruptions	▼	▲ (1)		▲ (1)
	Passivity during play	▲			▼ (1)
	Organised play	▲		▼ (1)	▼ (2)
	Complex play	▲		▼ (1)	▼ (2)

Notes: ^a^attention deficit hyperactivity disorder; ^b^cerebral palsy; ^c^developmental coordination disorder; ▲ depicts an increase in the play description; ▼depicts a decrease in the play description; values in parenthesis depict a number of studies.

## Data Availability

The data extraction tables used to support the findings of this study are included within the supplementary information file(s).

## Supplementary Materials

Supplementary Materials Supplementary Materials in the form of tables used in the data extraction process have been attached. Supplementary Table 1: table of articles rejected and the reasons. Supplementary Table 2: participant demographics, observational context and eligibility criteria. Supplementary Table 3: study aim, design, quality, outcome measures, and outcomes.
