# Supplementary material for: A Systematic Review Comparing the Play Profiles of Children with Special Health Care Needs with Typically Developing Children
Source: Biomed Res Int. 2020 Nov 19;2020:9582795. doi: 10.1155/2020/9582795 (PMC7695500; doi:10.1155/2020/9582795)
Supplement: Supplementary Materials — in the form of tables used in the data extraction process have been attached. Supplementary Table 1: table of articles rejected and the reasons. Supplementary Table 2: participant demographics, observational context and eligibility criteria. Supplementary Table 3: study aim, design, quality, outcome measures, and outcomes. [file 9582795.f1.docx]

**Supplementary Table 1: Table of articles rejected and the reasons**

| **Article** | **Study population** | **Play as outcome measure** | **No Comparison group at all** | **Comparison with another group not typically developing children** | **No full text in English** |
| --- | --- | --- | --- | --- | --- |
| Pfeifer, L.I., et al., Pretend Play of Children with Cerebral Palsy. Physical & Occupational Therapy in Pediatrics, 2011. 31(4): p. 390-402. |  |  | 1 |  |  |
| Cress, C.J., K.B. Arens, and A.K. Zajicek, Comparison of Engagement Patterns of Young Children with Developmental Disabilities between Structured and Free Play. Education and Training in Developmental Disabilities, 2007. 42(2): p. 152-164. |  |  | 1 |  |  |
| Sigafoos, J., D. Roberts-Pennell, and D. Graves, Longitudinal assessment of play and adaptive behaviour in young children with developmental disabilities. Research in Developmental Disabilities, 1999. 20(2): p. 147-162. |  |  | 1 |  |  |
| De Falco, S., et al., Fathers' play with their Down Syndrome children. Journal of Intellectual Disability Research, 2008. 52(6): p. 490-502. | 1 |  |  |  |  |
| De Falco, S., et al., Qualità della relazione affettiva madre-bambino e abilità di gioco in bambini con Sindrome di Down. = Quality of affective mother-child relations and game play ability in children with Down's syndrome. Infanzia e Adolescenza, 2008. 7(3): p. 124-137. |  |  | 1 |  |  |
| De Falco, S., et al., Mothers and Fathers at Play with Their Children with Down Syndrome: Influence on Child Exploratory and Symbolic Activity. Journal of Applied Research in Intellectual Disabilities, 2010. 23(6): p. 597-605. | 1 |  |  |  |  |
| Fewell, R.R., et al., The relationship between play and communication skills in young children with Down syndrome. Topics in Early Childhood Special Education, 1997. 17(1): p. 103-118. |  |  | 1 |  |  |
| Ryalls, B.O., et al., A perceptual motor intervention improves play behavior in children with moderate to severe cerebral palsy. Frontiers in Psychology, 2016. 7. |  |  | 1 |  |  |
| Venuti, P., et al., Play and emotional availability in young children with down syndrome. Infant Mental Health Journal, 2008. 29(2): p. 133-152. |  |  | 1 |  |  |
| Graham, N., J. Truman, and H. Holgate, An exploratory study: expanding the concept of play for children with severe cerebral palsy. British Journal of Occupational Therapy, 2014. 77(7): p. 358-365. |  |  | 1 |  |  |
| Johnson-Glenberg, M.C. and R.S. Chapman, Predictors of parent-child languange during novel task play: A comparison between typically developing children and individuals with Down syndrome. Journal of Intellectual Disability Research, 2004. 48(3): p. 225-238. |  | 1 |  |  |  |
| Ensor, R., et al., Gender differences in children's problem behaviours in competitive play with friends. Br J Dev Psychol, 2011. 29(Pt 2): p. 176-87. |  | 1 |  |  |  |
| Cordier, R., et al., Comparison of the play of children with attention deficit hyperactivity disorder by subtypes. Aust Occup Ther J, 2010. 57(2): p. 137-45. |  |  |  | 1 |  |
| Joginder Singh, S., T. Iacono, and K.M. Gray, An Investigation of the Intentional Communication and Symbolic Play Skills of Children with Down Syndrome and Cerebral Palsy in Malaysia. Journal of Early Intervention, 2014. 36(2): p. 71-89. |  |  |  | 1 |  |
| Sarimski, K., Early play behaviour in children with 5p- (Cri-du-Chat) syndrome. Journal of Intellectual Disability Research, 2003. 47(2): p. 113-120. |  |  |  | 1 |  |
| Rigby, P. and S. Gaik, Stability of playfulness across environmental settings: a pilot study. Phys Occup Ther Pediatr, 2007. 27(1): p. 27-43. |  |  |  | 1 |  |
| Cairney, J., et al., Developmental coordination disorder, age, and play: A test of the divergence in activity-deficit with age hypothesis. Adapted Physical Activity Quarterly, 2006. 23(3): p. 261-276. |  | 1 |  |  |  |
| Cairney, J., et al., Developmental coordination disorder, generalized self-efficacy toward physical activity, and participation in organized and free play activities. Journal of Pediatrics, 2005. 147(4): p. 515-520. |  | 1 |  |  |  |
| Cazeiro, A.P.M. and J.F.B. Lomônaco, Formação de conceitos por crianças com paralisia cerebral: Um estudo exploratório sobre a influência de atividades lúdicas. = Concept development by children with cerebral palsy: A study about the influence of playful activities. Psicologia: Reflexão e Crítica, 2011. 24(1): p. 40-50 |  |  |  |  | 1 |
| Lieber, J., A comparison of social pretend play in young children with and without disabilities. Early Education and Development, 1993. 4(3): p. 148-161. |  |  |  |  | 1 |
| Kennedy, M.D., et al., Play-language relationships in young children with developmental delays: implications for assessment. J Speech Hear Res, 1991. 34(1): p. 112-22. |  |  |  |  | 1 |
| Lalueza, J.L. and A. Perinat, Desarrollo de los significados compartidos en el juego entre adultos y niños con sï1drome de Down. = Development of shared meanings in play between adults and children with Down syndrome. Infancia y Aprendizaje / Journal for the Study of Education and Development, 1994. 67-68: p. 133-146. |  |  |  |  | 1 |
| Ogura, T., A. Notari, and R. Fewell, The relationship between language and play in Down syndrome children. Japanese Journal of Developmental Psychology, 1991. 2(1): p. 17-24. |  |  |  |  | 1 |
| Landau, R., et al., Parenting of 7-month-old infants at familial risk for ADHD during infant's free play, with restrictions on interaction. Infant Behavior and Development, 2009. 32(2): p. 173-182. |  |  |  | 1 |  |
| Aziz, S., et al. (2017). "Evaluate the Effectiveness of Play Activities on Inattention, Hyperactvity, Impulsivity and Anxiety in ADHD Children of Age Group 6-12 yrs." Indian Journal of Physiotherapy & Occupational Therapy 11(4): 208-211 |  |  |  |  | 1 |
| Fleming, A. P., et al. (2017). "Structured Parent-Child Observations Predict Development of Conduct Problems: the Importance of Parental Negative Attention in Child-Directed Play." Prev Sci 18(3): 257-267. |  |  | 1 |  |  |
| Miller, L. J., et al. (2017). "Play in Natural Environments: A Pilot Study Quantifying the Behavior of Children on Playground Equipment." Journal of Occupational Therapy, Schools & Early Intervention 10(3): 213-231. |  |  | 1 |  |  |
| Harbourne, R. T., et al. (2018). "Sitting Together And Reaching To Play (START-Play): Protocol for a Multisite Randomized Controlled Efficacy Trial on Intervention for Infants With Neuromotor Disorders." Physical Therapy 98(6): 494-502. |  |  |  |  | 1 |
| Swank, J. M. and S. Smith-Adcock (2018). "On-task behavior of children with attention-deficit/hyperactivity disorder: Examining treatment effectiveness of play therapy interventions." International Journal of Play Therapy 27(4): 187-197. |  |  |  | 1 |  |
| , ب., et al. (2018). "مقایسه اثربخشی بازی درمانی و قصه گویی بر بهبود نشانگان اختلال نقص توجه/بیش فعالی دانش آموزان." Qom University of Medical Sciences Journal 12(8): 59-68. |  |  |  |  | 1 |
| Borg, S. (2009). ADHD and problem-solving in play. Emotional and Behavioural Difficulties, 14(4), 325-336. |  |  |  | 1 |  |
| **Total** | 2 | 4 | 10 | 7 | 8 |

**Supplementary Table 2: Participant demographics, observational context and eligibility criteria**

| **Article** | **Participants** | **Playmate/s** | **Diagnosis/**  **Condition** | **Observational Context** | **Eligibility Criteria** |
| --- | --- | --- | --- | --- | --- |
| Alessandri (1992) | **Sample size per group:**  20 ADHD +20 without ADHD  **Age Range:** 4-5years old  **Mean Age:**  ***Experimental group***: 52.6 months  ***Control group:*** 53.3 months  **Gender:**  ***Experimental group:*** 17 boys and 3 girls  ***Control group:*** 17 boys and 3 girls | **Number of playmates:**  NR  **Relationship:**  Classmates | **Experimental group:**  ADHD  **Control group:** Typically developing | ***Setting*:** Classroom  ***Observation time***: 60 min on each child | **Experimental group:**  **ADHD:** (a) complaints of short attention span, impulsivity, and hyperactivity at home as reported by mothers;  (b) problems persisted for 6 months;  (c) behaviour that met DSM-III-R classification for ADHD and no other disorder;  (d) a T-score greater than the 84th percentile on the hyperactivity index on the Conners Abbreviated Teacher Questionnaire (CATQ; Goyette, Conners  (e) IQ of 80 or higher on the Stanford-Binet Intelligence Scale (4^th^ ed); and  (f) No history of treatment with stimulant drugs.  **Control group:**  (a) no parent complaints of significant behavior problems,  (b) no history of ADHD as documented by clinical interview,  (c) a score below the 84th percentile on the CATQ  (d) an IQ of 80 or higher. |
| Cordier, Bundy, Hocking, and Einfeld (2010b) | **Sample size per group** 112 with ADHD + 126 typically growing children  **Age Range**: 5-11years  ***Mean Age:***  ***Experimental group:*** *106.8 months*  ***Control group:*** *103.2 months*  **Gender:**  ***Experimental group:*** 80.3% Boys and 19.7% Girls  ***Control group:*** 78.7% Boys and 21.3% Girls | **Number of playmates:**  one child with ADHD and one typically developing child in each observation  **Relationship:** playmate familiar to child (60% of the playmates of children with ADHD were siblings because that proportion of the children with ADHD identified that they did  not have another usual playmate) | **Experimental group:**  ADHD  **Control group:** Typically developing | ***Setting:*** ***Experimental group:*** playroom in a clinical setting  ***Control Group:***  a designated play area at the respective schools  ***Observation time:*** 20minutes | **Experimental group:**   1. Children had a formal diagnosis of ADHD made by a psychiatrist or paediatrician according to DSM-IV criteria. 2. had conditions known to be comorbid to ADHD, such as learning disorders, oppositional defiant disorder, conduct disorder, anxiety disorder, and mood disorder, provided that ADHD was the primary diagnosis 3. children with ADHD who were receiving the short-acting forms of methylphenidate given that their duration of action is 3 to 5 hours (American Academy of Child and Adolescent Psychiatry, 2007; Physicians’ Desk Reference, 2007).   **Control group:**  Child who did not have ADHD (i.e., scored below the clinical cut-off for any of the Conners’ Parent Rating Scales–Revised  [CPRS-R] subscales and DSM-IV scales) and for whom no concerns had been raised about development by a teacher or health professional. |
| Landau, Amiel-Laviad, Berger, Atzaba-Poria, and Auerbach (2009) | **Sample size per group:** 59  34 boys in the risk group and 25 boys in the comparison group  **Age Range**  ***Experimental group:*** 6.9 -7.36 months  Control group: 7.63-8.12 Months  **Mean Age:**NR  **Gender**: all boys  ***Experimental group:***  ***Control group:*** | **Number of playmates:** No Playmates  **Relationship:**  N/A | **Experimental group:** ADHD  **Control group:**  Children without ADHD | ***Setting*: Experimental group:** Laboratory room  **Control Group**:  Laboratory room  ***Observation time*:** NR | Experimental group: a score of ≥7, and for recruitment into the comparison group  Control Group: a score of ≤3, with background characteristics similar to the risk group.  **Exclusion Criteria:**  Infants with a history of prenatal, perinatal or postnatal complications, including low birth weight and prematurity, were excluded. |
| (Okimoto, Bundy, & Hanzlik, 2000) | **Sample size per group:**  19 children with cerebral palsy and developmental delays + 19 children with no cerebral palsy or developmental delays.  **Age Range:**  ***Experimental group:*** 8-32 months  ***Control group:***3-18 months  ***Mean Age:***  ***Experimental group:***18 months  ***Control group:***9 months  **Gender:**  ***Experimental group:*** 13 Boys and 6 girls  ***Control group:***13 Boys and 6 Girls | **Number of playmates:**  Only the mother present  **Relationship:**  Mother | **Experimental group:**  Children with cerebral palsy and developmental delays  **Control group:**  Children without cerebral palsy and developmental delays | ***Setting***: home setting  ***Observation time:*** 15-min free play session | **Experimental group:** Not clearly defined. participants who were videotaped for Hanzlik’s (1986) study were used for the current research purpose of scoring the playfulness of young children using the ToP.  **Control group:** Not clearly defined. |
| (Hsieh, 2012) | **Sample size per group**  30 children with CP +  29 age-matched typically developing children  **Age Range:**  ***Experimental group:*** 72-96 month  ***Control group:***72-96 months  ***Mean age:***  ***Experimental group:*** 88.08 months  ***Control group:*** 88.08 months  **Gender:**  ***Experimental group:*** 16 boys 13 girls  ***Control group:***15 boys and 14 girls | **Number of playmates:** NR  **Relationship:**  NR | **Experimental group:** Children with cerebral Palsy  **Control group:** Typically developing children | ***Setting:*** Room designed for free play sessions at each school.  ***Observation time:***10-min Individual sessions, designed twice a week to occur over a period of ten weeks. | **Experimental group:**  -CP children having moderate to severe fine motor problems as indicated by PDMS-2.  -Large motor problems of these subjects included the inability to walk, jump, and abnormal muscle tone.  -Muscle tightness influenced the arms in subtle ways.  -They also had trouble lifting heavier objects with a certain hand or difficulty grasping objects, holding a pencil, or manipulating objects.  -To prevent children with CP have additional intellectual impairments that may affect pretend, all subjects were reported by their teachers as having passed the cognitive components of developmental screen.  **Control group:**  All control participants were reported by their teachers to be healthy and to display normal learning skills. |
| (File, 1994) | **Sample size per group.** 14 had mild or moderate cognitive and/or speech and language delays. + 14 typically growing children  **Age Range:**  **Experimental group:** 56 months  **Control group:** 56 months  **Mean Age: Experimental group**: 33-73 months;  **Control group:** 33-73 months  **Gender:**  ***Experimental group:*** 24 boys and 4 girls  ***Control group:***24 boys and 4 girls | **Number of playmates:** NR  **Relationship:**  NR | **Experimental group:** Had mild or moderate cognitive and/or speech and language delays  **Control group:** Typically growing children | ***Setting:*** Classroom  ***Observation time:*** two 45-minute visits to each classroom during free play periods. | **Experimental group:** 14 had mild or moderate cognitive and/or speech and language delays. children with physical disabilities and/or severe disabilities were not included in the study, in order to reduce heterogeneity of the sample  **Control group:** Each child with disabilities was matched with a typically developing peer from the same classroom who was the same sex and approximately the same age. |
| (Hestenes & Carroll, 2000) | **Sample size per group:**  8 had disabilities + 21 typically developing  **Age Range:**  **Experimental group:** 41-76 months  **Control group:** (not stated)  **Mean Age:**  ***Experimental group:*** 57.8 months  ***Control group****:* 53.6 months  **Gender:**  ***Experimental group:*** 6 boys 2 girls  ***Control group:*** 12 boys and 9 girls | **Number of playmates: NR**  **Relationship:** classmates | **Experimental group:** Children with disabilities  **Control group:** Typically developing | ***Setting:*** classrooms and outdoor yards at school.  ***Observation time:*** observers watched each area of the classroom or play yard containing one or more children for 10 s and then recorded the appropriate information on the map during the next 20 to 30 seconds. | **Experimental group:** not clearly stated  **Control group:** not clearly stated |
| (Skinner, Buysse, & Bailey, 2004). | **Sample size per group:** 55 Children with mild to moderate developmental delays + 110 play partners with disabilities + 110 play partners without disabilities  **Age Range:**  ***Experimental group:*** *33-60 months*  ***Control group with disabilities:***33-61 months  ***Control group without disabilities:*** 35-57 months  **Mean Age:**  **Experimental group:** 48.99 months  **Control with disabilities:** 47.85 months  **Control without disabilities:** 46.57 months  **Gender:**  ***Experimental group:*** 12 girls and 43 boys  ***Control with disabilities:*** 24 girls and 86 boys  **Control without disabilities:** 24 girls and 86 boys | **Number of playmates**: four different same-sex partners in a standardised  dyadic play  situation  **Relationship:**  All familiar to the play mate | **Experimental group:** preschool children with mild to moderate developmental delays.  **Control group:**  110 play partners with disabilities and 110 play partners without disabilities | ***Setting:*** Outside the classroom in a specially designed play area.  ***Observation time:*** two, 15-min sessions with each of the four playmates | **Experimental group**:  -All were eligible for special education services under provisions of Part B-Section 619 of the Individuals with Disabilities Act (IDEA; 1999).  -Socio-economic status of these children’s families varied from 17 to 66 on the Hollingshead Index (Hollingshead, 1975), with a mean of 44.  **Control group:** All of the children were enrolled in some type of inclusive program, including a university-based inclusive childcare program, a community-based integrated developmental day program.  **-**Partners were selected from a list of children with a full range of chronological and developmental ages that were normally distributed around their means**.** |
| (Cairney, Kwan, Hay, & Faught, 2012) | **Sample size per group**  (Total of 590), A total of 44 children with DCD and 546 children typically growing children  **Age Range:**  ***Experimental group:*** not stated  ***Control group:***  108 to 168 months,  ***Mean Age:***  ***Experimental group:*** Not stated  ***Control Group:*** 137.52 (SD = 1.46).  **Gender:**  ***Experimental group:*** 25 girls and 19 boys  ***Control group:*** NR | **Number of playmates:** NR  **Relationship:**  N/A | **Experimental group:** DCD  **Control group:** Typically growing children | ***Setting:*** NR  ***Observation time:*** NR | **Experimental group:**  All students in grades four through eight from five elementary schools in the Niagara Region of Ontario,  Canada.  Eighteen children with pre-existing physical limitations, excluded from physical education classes due to medical reasons, were excluded from the study.  Eight children with previously known learning disorders were allowed to take part in the study but were excluded from the analyses  **Control group:**  All students in grades four through eight from five elementary schools in the Niagara Region of Ontario,  Canada. |
| (Smyth & Anderson, 2000) | **Sample size per group**  55 in the DCD + 55 typically developing  ***Age range:***NR  ***Mean Age: NR***  **Gender:**  ***Experimental group:*** 38 boys and 17 girls  ***Control group:***  *38 boys and 17 girls* | **Number of playmates:** Number of playmates varied from playing alone, with 1 other, 2 others, 3 others, 4 others, 5–7 others or with 8+ others**.**  **Relationship:**  Classmates | **Experimental group:**55 in the DCD  **Control group:** 55 typically developing | ***Setting:*** classroom, playground  ***Observation time:*** Each observation period lasted 5 min and was broken into 30-s intervals. | **Experimental group:** Thirty children in the DCD group scored below the 5th centile on the Movement ABC and 25 scored between the 5th and 15th centile  -Children who had been identified as having special educational needs in addition to, or other than, clumsiness were not included  -Children diagnosed as having special needs in relation to Asperger’s syndrome, general developmental delay and attention deficit disorder were excluded.  **Control group:**  Matched on gender, age and verbal ability |
| (Venkatesan & Ravindran, 2012) | **Sample size per group**  42 children with developmental disabilities + 42 siblings  **Age Range:NR**  ***Mean Age***  ***Experimental group:*** (129.6 months) and girls (97.2 months)  ***Control group***: boys (138 months) and girls (139.2 months)  **Gender:**  ***Experimental group:***23 boys and 19 girls  ***Control group:*** 17 boys and 25 girls | **Number of playmates:** Varied (depended on the number of siblings in family, available for the study).  **Relationship:**  siblings | **Experimental group:**  Children with Developmental disabilities which include mental retardation (14), hearing loss (8) and others (20). The others included speech delays, autism, fluency disorders, multiple disabilities, children with emotional and conduct disorders.  **Control group:**  Typically developing siblings | ***Setting:*** NR  ***Observation time:*** NR | **Experimental group:** Children with developmental disabilities attending Therapy clinics at all India Institute of Speech and Hearing, under the ministry of Health and family Welfare, Government of India, Mysore.  -Each individual underwent an assessment through case history and diagnostics assessment which combined opinions from specialist like ENT, neurology, Clinical Psychology, physiotherapy, occupational therapy, audiology and speech language pathology.  **Control group:**  Offspring sharing the same biological origins and resident members of their natural home settings and those who have continually lived together under same roof as one family along with their parents or extended family members as the case could be. |
| (Linn, Goodman, & Lender, 2000) | **Sample size per group**  14 with Down syndrome + 14 typically developing children  **Mean Age:**  ***Experimental group:*** 29.10 Months  ***Control group:*** 28.70 Months  **Age Range: NR**  **Gender:**  ***Experimental group***: 7 girls and 7 boys  ***Control group:*** 7 girls and 7 boys | **Number of playmates:** Child playing alone, but mother and the research assistant are present in room  **Relationship:**  N/A | **Experimental group:** 14 with Down syndrome  **Control group:** 14 typically developing children | ***Setting:*** standardised playroom  ***Observation time:*** The play session lasted from 30 to 60 minutes depending upon the child’s involvement with the toys | **Experimental group:**  -a developmental age between 20 and 40 months, as assessed with the Mental Scales of the Bayley Scales of Infant Development (Bayley, 1993), and --the ability to sustain involvement in the play session for at least 30 minutes (remain in the play room and not indicate verbally or non-verbally the desire to terminate the session). All children were from Caucasian, English-speaking families and lived with at least one biological parent.  **Control group:**  Matching individual subjects with subjects from the group of children with Down syndrome on developmental age (+/-2 months), sex, ethnicity, and mother’s level of education. |
| (Pearton, Ramugondo, Cloete, & Cordier, 2014) | **Sample size per group**  15 children who had positive histories of Prenatal Alcohol Exposure (PAE) + 15 Typically developing children.  **Age Range: NR**  **Mean Age (years): *Experimental group:***84.6 (0.50)  ***Control group:*** 81.72 (0.58)  **Gender:**  ***Experimental group:***8 boys and 7 girls  ***Control group:***7 boys and 8 girls | **Number of playmates:** NR  **Relationship**: N/A | **Experimental group:** 15 children who had positive histories of Prenatal Alcohol Exposure (PAE)  **Control group:**  15 Typically developing children. | ***Setting:*** School playground  ***Observation time:*** 15 minutes | **Experimental group:** Participants needed to have a history of minimal to heavy PAE, Children were excluded from the study if they had histories of exposure to other drugs in utero, except nicotine.  -Children were also excluded from the study if they currently or previously received treatment or play or socio-behavioural problems.  -Children were excluded if they had known major neurodevelopmental or psychiatric disorders, such as Autism or cerebral Palsy  **Control group:** This group included 15 children who did not have a history of PAE. No history of PAE was confirmed by parents or guardians during a semi-structured interview discussing the children’s developmental history. |
| (Malone, 2009) | **Sample size per group**  17 children with intellectual disabilities + 17 typically growing children  **Age Range:** NR  **Mean Age:**  ***Experimental group:*** 52.35 months (SD = 14.01  ***Control group:*** CA of 27.76 months (SD = 11.37)  **Gender:**  ***Experimental group***: 10 boys and 7 girls  ***Control group:*** 10 boys and 7 girls | **Number of playmates:** child played independently  **Relationship:** N/A | **Experimental group:** 17 children with intellectual disabilities  **Control group:** 17 typically growing children | ***Setting:*** home-based independent play situation and a classroom-based free play situation.  ***Observation time:*** 30-minute video | **Experimental group:** All children were recruited from Patterns of Home- and Classroom-based Toy Play 335 seven inclusive preschool programmes (i.e., general education programmes into which children with developmental concerns were enrolled).  **Control group:**  Groups were matched, child-by-child, on cognitive developmental age (DA) and gender. |
| (Hamm, 2006) | **Sample size per group**  20 children with disabilities + 20 children without disabilities  **Age Range:NR**  **Mean Age:**  ***Experimental group:*** 16.3 months  ***Control group:*** 16.2 months.  **Gender:**  ***Experimental group:***14 boys and 6 girls  ***Control group:*** 13 boys and 7 girls | **Number of playmates:** Caregivers were instructed to engage in typical play  with their child**,** (one caregiver)  **Relationship:**  caregiver | **Experimental group:** 20 children with disabilities. Types of disabilities varied, and ranged from mild to severe limitations  **Control group:**  20 children without disabilities | ***Setting:*** child’s natural play environment***.***  a room in the family home where the child typically engaged in play  ***Observation time:***  a 20-minute | **Experimental group:** All families had a socioeconomic status of working or middle class and all children, with the exception of one boy with a developmental disability, resided with both parents.  -All participating parents had completed high school and most were college educated (15 parents of children without disabilities and 14 parents of children with disabilities).  -All fathers in both groups were employed outside the home. Nine mothers of children with disabilities worked outside  -The home, whereas 16 mothers of children without disabilities worked outside the home.  **Control group:** |
| (Angelin, Sposito, & Pfeifer, 2018) | **Sample size per group**  30 children with CP and 30 children with TD,  **Age Range:** aged 36–72 months (with a tolerance of four months after the sixth birthday  **Mean Age:**  ***Experimental group:*** *Mean* 54.6months SD 12.4  ***Control group:*** *Mean* 53.9months, SD10.9  **Gender:**  ***Experimental group:*** *19 boys and 11 girls* ***Control group:*** 15 boys and 15 girls | **Number of playmates:**  NR  **Relationship:** | **Experimental group:**  30 children with CP  **Control group:**  30 children with Typically Developing, | ***Setting:***  Private room, provided by the rehabilitation centre (for children  with CP) or at school (for children with TD), without the interference of external stimuli.  ***Observation time:*** NR | **Experimental group:** children with CP were;  (1) diagnosed with CP, (2) age 3–6 years, (3) able to understand  simple commands, and (4) consented by parents to participate in this study.  **Control group:** The group of children with TD was compared to the group of children with CP in terms of age and sex. |
| (Wilkes-Gillan, Bundy, Cordier, Lincoln, & Chen, 2016) | **Sample size per group**  5 children with ADHD and 5 playmates  **Age Range:NR**  **Mean Age:** 19 months; SD; 0.8 for all participants  ***Experimental group:***  ***Control group:***  **Gender:**  ***Experimental group:*** *4 boys and 1 girl*  ***Control group:*** *2 boys and 3 girls* | **Number of playmates:** 5  **Relationship:** 4 were siblings and 1 was a friend | **Experimental group:** 5 children with ADHD  **Control group:**  5 playmates | ***Setting:*** clinic playroom  ***Observation time:*** 20 mins | **Experimental group:** Three children with ADHD presented with the inattentive subtype with T-scores above the clinical cut-off (>70) on the inattention subscale (mean group inattention scale T-score=74.3; SD;7.9). Two children with ADHD presented with the hyperactive subtype with T-scores above the clinical cut off (>70) on the hyperactive scale subtype (mean group hyperactive scale T-score¼64; SD¼11.2)  **Control group:** Playmates had T-scores below the borderline clinic cut-off (<65) on all symptom scales. |
| (Barnes, Wilkes-Gillan, Bundy, & Cordier, 2017). | **Sample size per group**  13 children with ADHD and 13 TD children  **Age Range:NR**  **Mean Age:**  ***Experimental group:*** *120 months SD (2.1)*  ***Control group:*** *111.6 months SD (2.3)*  **Gender:**  ***Experimental group:*** *11 boys and 2 girls*  ***Control group:*** *6 boys and 7 girls* | **Number of playmates:** 13  **Relationship:** 8 of the 13 playmates were siblings | **Experimental group: 13 children with ADHD**  **Control group:**  **13 TD children** | ***Setting:*** home environment and in the clinic  ***Observation time:*** 20 minutes | **Experimental group:** Children with ADHD who participated in the 10-week RCT intervention 12 months earlier (Wilkes-Gillan et al., 2016) were invited to participate in the study with a playmate.  -Participating children’s level of ADHD symptoms were measured using the Conners Comprehensive Behavior Rating Scales (CCBRS), with a T score ≥65 on one ADHD DSM-IV subscale indicating clinical level of symptoms**.**  **Control Group:** Playmates were required to be of similar age and either a peer, sibling or cousin who regularly spent time with the child with ADHD. |

*Notes*. Abbreviations: M = Mean; SD = Standard deviation; TD = typically developing; NR = not reported; N/A = not applicable;

Setting refers to place/location were data collection was done; Observation time refers to time taken by researchers observing the children whilst engaged in play

**Supplementary Table 3: Study aim, design, quality, outcome measures and outcomes**

| **Study** | **Study aim** | **Study Design** | **Quality of the studies (KMET)** | **Primary measures used to measure play and secondary**  **outcome measure/tools used** | **Findings on play/playfulness** | **Other findings** |
| --- | --- | --- | --- | --- | --- | --- |
| Alessandri (1992) | To observe the play and non-play behaviours of ADHD and non-ADHD preschool-age children in a free play situation. | Cross sectional  study | 76.9% | **Primary outcome measure:** Quality of social participation and level of cognitive play Rubin (1989).  Used adapted tool, (actual name not mentioned  **Secondary Outcome Measure:** non-play categories were coded, tool not mentioned clearly | ADHD children engaged in less dramatic play compared with non-ADHD children (M = 413.35 vs. 643.25, p < .05). Although there were no differences in the frequency of solitary play between the two groups, ADHD children engaged in less parallel (M =912.25 vs. 1330.25) and less group play (M = 162.05 vs. 294.75, all p < .01) compared with non-ADHD children. | ADHD children engaged in greater negative affective exchanges  when with adults compared with non-ADHD children (M = 62.75  vs. 12.50, F (1, 38) = 12.64, p < .01). Moreover, ADHD children engaged in fewer peer conversations than non-  ADHD children, F (1, 38) = 8.64, p < .01. |
| Cordier et al. (2010b) | To examine the similarities and differences in play behaviour of children as having ADHD and typically developing children. | Cross sectional study |  | **Primary outcome measure:**  ToP (Bundy, 2004)  **Secondary Outcome Measure**  Conners’ Parent Rating Scales–Revised [CPRS-R] subscales (Hale, How, Dewitt, & Coury, 2001) | Children with ADHD were less playful than the typically developing children (ADHD mean measure score = 1.09; ADHD standard deviation = 1.28; Control mean measure score = 1.99; control standard deviation = 0.82; t = -13.9; p < .01; df = 125). | Children with ADHD performed significantly more poorly on five of the eight social items: shares (4); support (5); intensity (11) and skill of social play (12); and skill in responding to cues (29) (Table 3). However, of the remaining three social items, children with ADHD performed significantly better than typically developing children on skill to negotiate (2). There was not a significant difference for the skill to initiate (1) or extent of social play (10). |
| Landau et al. (2009) | -To determine if there were differences in patterns of interaction of mothers and fathers with their 7-month-old boys at familial risk for ADHD | Longitudinal Study | 73% | **Primary outcome measure:**  Coding  **Secondary Outcome Measure**  Laboratory Temperament Assessment  Battery (LAB-TAB) (Goldsmith & Rothbart, 1996),  DSM-IV (American Psychological Association, 1994)  Tridimensional Personality Questionnaire (Cloninger, 1987)  Conners Adult ADHD Rating Scale (CAARS; Conners, Erhardt, & Sparrow,  1998) | No differences for Needs Help were found between groups (The experimental and the control group), F (1,56) = .89, p= .35, ᶇᶈ²= .016, or between the infant’s behaviour in the presence of the mother and in the presence of the father, F(1,56) = 1.04, p = .31, ᶇᶈ² = .018. Nor was the interaction between group and parent significant, F(1,56) = .02, p = .90, ᶇᶈ² = .000.  The percentage of infants who tried to involve their father was significantly higher than the percentage who tried to involve their mother, = 46.99, df=1, p < .001. | No differences were found for group or for parent in the ANOVA analysis. Nor was the interaction between parent and group significant. However, when analyzed separately, the between-group difference for mother’s behavior was significant, t(45) =−2.34, p = .02, g = .569. |
| Okimoto et al. (2000) | -To determine whether  the ToP (Version 3) was a reliable and valid instrument  to assess young children.  -To compare the playfulness of young children with cerebral palsy and developmental  delays with that of peers who are typically  developing  -To compare the  effect of an intervention aimed at improving mother–infant interactions with that of neurodevelopmental treatment (NDT) on the children’s playfulness. | Quasi-experimental design | 84.6% | **Primary outcome measure:**  The ToP, Version 3 (Bundy, 1997b)  **Secondary Outcome Measure** N/A | The mean score of the children who were typically developing was significantly higher on the ToP than the mean score of their matched counterparts with cerebral palsy and developmental delays (t = –3.938, p = .0005). | There was no significant difference between the mean gain score of the children whose mothers received the intervention and that of the children who received NDT (t = .562, p = .583). |
| Hsieh (2012) | -To examine affective expressions and imagination in children with CP as a function of ordinary versus adaptive pretend play. | Quasi-experimental design | 84.6% | **Primary outcome measure:**  Play Scale-Brief Rating (APS-BR) (Sacha Cordiano, Russ, & Short, 2008).  **Secondary Outcome Measure:**  Fine Motor Scale of the Peabody Developmental Motor Scales, 2nd edition (Folio & Fewell, 2000) | Multivariate analysis of variance (MANOVA) was use to investigate the relationship between Child Group (CP and typically developing children) and Play Type (adaptive and ordinary pretend play). Children with CP showed significantly more affective expression for adaptive pretend play than for ordinary pretend play, F(1, 28) = 96.475, p = 0.000, ᶇᶈ²= 0.775.  The results show more significant positive changes of imagination behaviors occurring in children with CP during adaptive pretend play, F(1, 28) = 107.205, p = 0.000, ᶇᶈ² = 0.793. | The mean scores for the APS-BR in the CP group increased across adaptive pretend play sessions as hypothesized (F = 192.783, p = 0.000, ᶇᶈ²= 0.043).  Analyses illustrated that play type differences appeared to influence the emotion expression and imagination of CP children during the pretend play session. |
| File (1994) | -To examine the cognitive and social complexity of  their play and their interactions with teachers. | Cross sectional study | 76.9% | **Primary outcome measure:** Coding tool based upon the Rubin, Maioni, and Hornung (1976) adaptation of the work of Parten and Smilansky.  **Secondary Outcome Measure:**  -The Peer Relation Rating Scale was adapted from Ladd and Muth (1990).  -Assessment of Teacher Role Scale developed for this study consisted of 6 vignettes. | The proportion of time children engaged in play activities was identical for children with disabilities and developing typically (70%).  The 28 participants spent an average of 56% of their play at the functional level (range =.18-.98). | -The main effect for children’s developmental status was significant, F(1, 25), p= 9.41, ᶇᶈ²= .005.  -Children were uninvolved with teachers for two thirds (67%) of the time.  -T-tests revealed no differences  in how often teachers were uninvolved, involved in routines,  or watched children according to the children’s developmental status. |
| Hestenes and Carroll (2000) | -To describe the context of play as well as to better understand the experience of inclusive classrooms for children with and without disabilities, young children’s  play interactions and beliefs in inclusive preschool settings were also examined | Cross sectional study | 88.5% | **Primary outcome measure:**  scan-sampling technique (Nabors, 1995)  **Secondary** Outcome Measure: Competency Ratings for Disabilities (Diamond, 1994; Diamond & Hestenes, 1996; Diamond et al., 1997) | Typically developing children spent over half their time in cooperative play. Children with disabilities spent about one third of their time in cooperative play and one third of their time in solitary play. | -Typically developing children were significantly less likely to interact with their peers with disabilities (14.3%) than would be expected (29%).  -A paired-sample t test showed that children with disabilities also spent significantly less time interacting with their typically developing peers (33%) than was expected (71%; t(8) 5.83, p.01).  -There were no differences in typically developing children’s reported preferences to play with peers without disabilities (M 2.07, SD 0.37) versus peers with disabilities (M 2.04, SD 0.49) |
| Skinner et al. (2004) | -To examine how total duration of social play of preschool children with disabilities varied as a function of Both chronological and Developmental age of their social partners. | Cross sectional study | 66.7% | **Primary outcome measure:**  Parten’s social play categories.  Parten’s (1932)  **Secondary Outcome Measure:**  Behavioral observations conducted were using Observer 3.0 for Windows, with the Video Tape Analysis (Noldus Information system Technology, 1996).  Battelle Developmental Inventory. The Battelle Developmental Inventory (BDI; Newborg, Stock, Wnek, Guidubaldi,& Svinicki,1988) | Children with lower developmental ages displayed less associative play with more advanced partners.  A child at a more advanced developmental level (developmental age of 45 months), however, would exhibit longer total duration of associative play with partners of more advanced development than of  Partners with less advanced developmental (B = 6.23, age effect size = .23). | Focal child with developmental age of 34 months would not exhibit different age durations of associative play with partners different of developmental levels(B =- .69, effect size= .025). |
| Cairney et al. (2012) | To investigate the effect of gender on the relationship between Developmental Coordination Disorder (DCD) and self-reported participation in organized and recreational free-play activities | Cross sectional study | 86.3% | **Primary outcome measure:**  The Participation  Questionnaire (PQ)  (Hay, 1992)  **Secondary Outcome Measure**  Participation Questionnaire Children with DCD reported lower self-efficacy toward physical activity (M = 62.16, SD = 10.52) than did children without DCD (M = 48.30, SD = 11.90).  The Children’s Self-perception of Adequacy in and Predilection for Physical Activity (CSAPPA) scale (Hay, 1992).  Short form Bruininks-Oseretsky Test of Motor Profi ciency (BOTMP-SF; Bruininks, 1978). | Children with DCD (M = 13.79, SD = 4.22) were less likely than their motor proficient peers (M = 14.81, SD = 4.72) to engage in recreational free play activities. | Children with DCD reported lower self-efficacy toward physical activity (M = 62.16, SD = 10.52) than did children without DCD (M = 48.30,  SD = 11.90). |
| Smyth and Anderson (2000) | -To determine whether a child’s performance on a test of motor ability is related to later involvement in both social and physical activities in the school playground.  -To explore the relationship between the physical demands of play and the sizes of the groups in which it takes place and how this changes with age. | Cross sectional study | 87.5% | **Primary outcome measure:** Psion Workabout computer using a detailed coding scheme developed in the Observer software system. Boulton and Smith (1993)  **Secondary Outcome Measure**  Movement Assessment Battery for Children (the Movement ABC; Henderson & Sugden, 1992). | Most children spent their playtime in single sex  groups (F(2,106) = 524.4, p < 001).  Boys were observed in the less active group more often than in the more active group (F(1,74) = 30.47, p < .001), but that there was no significant interaction between these factors (F(1,74) = 1.96, p > .05). | There was a significant effect of group membership (F(1,102) = 7.34, p < .01), with children in the DCD group spending more time alone, no effect of age, and no interaction (F < 1 in both cases). In a separate analysis there was no reliable effect of gender F(1,106) = 1.59, P > .05) and no interaction between gender and group membership (F < 1). |
| Venkatesan and Ravindran (2012) | -To delve deep and specifically into play behaviours and activities between affected and unaffected siblings in relation to variables like age, gender, education, type of disability and number of siblings | Cross sectional exploratory research | 75% | **Primary outcome measure:** Play Activity Checklist for children with Mental Retardation (PACK-MR) (Khoshali and Venkatesan, 2010)  **Secondary Outcome Measure**  N/A | Children with developmental disabilities (42) manifest varieties of play behaviours like maintains eye to eye contact with peers (n: 37/42; 88.1%), shares own belongings/play materials with peers (n: 35/42; 83.3%). | Children attending special schools (N:23; Mean: 14.9; SD:4.7) as well as regular schools (N:16; Mean: 9.2; SD: 2.6) appear to have advantage and range of play behaviours compared to children with no school exposure (N:3; Mean: 1.1; SD: 0.7) F: 22.79; p<0.001). |
| Linn et al. (2000) | -To investigate how children with Down syndrome performed without the  typical  constraints of time and adult directives | Cross sectional study | 91.6% | **Primary outcome measure:**  Identification of play episodes followed procedures established by McCune Nicolich (1983).  **Secondary Outcome Measure:** coded based on categories determined by Krakow and Kopp (1983). | All subjects played for at least 30 minutes  Children with Down syndrome fell into two groups; short players (n=4) and long players (n=10). After 47 minutes, the 10 long players with Down syndrome discontinued play at a nearly uniform rate, until play for all children was stopped at 60 minutes.  Typically developing children first ended play at 33.4 minutes and the rate of discontinuation remained steady until play for all children was stopped at 60 minutes | Children with Down syndrome interrupted play more often to engage in social behavior than their typically developing mental-age-matched peers. Children with Down syndrome had an average of 1.36 social episodes (range 0–8), and typically developing children had an average of .71 social episodes (range0–4). |
| Pearton et al. (2014) | -To investigate the differences in playfulness  of children with and without prenatal alcohol exposure. | Cross sectional comparative study | 95.4% | **Primary outcome measure:**  Test of playfulness (ToP)  **Secondary Outcome Measure:**  Questionnaire was used to determine which children  met the inclusion and exclusion criteria as well as for  inter-group comparisons on demographic information | The PAE group were found to have significantly lower overall mean playfulness scores than the reference Group (t=2.51; df=28; p=0.02). | The PAE group was found to have  scored significantly lower on 5 of the 12 ToP items related to social play than their reference group counter parts.  Children with PAE were found to score significantly lower than the reference group on two other items  not exclusively related to social play, namely ‘Transitions’ from one play activity to another (t=7.74; df=1; P =0.01), and ‘Modifies’ activity to maintain challenge or  make the activity more fun (t=5.60; df=1; P=0.03) |
| Malone (2009) | -To analyse, the within and between group  patterns of play demonstrated by preschool children with and without intellectual disabilities  in home-based independent play and classroom-based freeplay situations are presented | Cross sectional study | 75% | **Primary outcome measure:**  Coded for categorical and sequential play  **Secondary Outcome Measure:**  Battelle Developmental Inventory (BDI; Newberg, Stock, Wneck, Guidubaldi, & Svinicki,  1984) | During the classroom-based free play situation children with intellectual disabilities spent more time engaged in constructive play than either functional play or pretend play. A statistically significant effect emerged for these children’s classroom based categorical play variables [F(1.25, 20) = 9.07, p < .01; ᶇᶈ² = .36].  During the home-based play situation children without intellectual disabilities spent more time engaged in constructive play than either functional play or pretend play. As demonstrated by children without intellectual disabilities was statistically significant [F(2, 32) = 3.84, p < .000; ᶇᶈ²=.60] | During the home-based play situation children with intellectual disabilities spent more time engaged in unordered multi-scheme play sequences than either single scheme play sequences or ordered multi-scheme play sequences.  During the classroom-based free play situation children with intellectual disabilities spent more time engaged in single scheme play sequences than either unordered multi-scheme play sequences or ordered multi-scheme play sequences.  The overall analysis of  length of play sequences was neither statistically significant nor meaningful [F(2, 32) =2.1.47, p .25;  = .08]. During the classroom-based free play situation children without  intellectual disabilities spent more time engaged in single scheme play sequences than either unordered multi-scheme play sequences or ordered multi-scheme play sequences. |
| Hamm (2006) | -To examine the reliability and validity of the Test of  Playfulness (ToP) and the Test of Environmental Supportiveness (TOES), to compare playfulness and environmental support of play in children with and without developmental disabilities, and to examine the correlation between the ToP and the TOES. | Cross sectional study | 92.3% | **Primary outcome measure:**  Test of Playfulness (ToP). The ToP (version 4.0)  (Bundy, 1991)  **Secondary Outcome Measure**  Test of Environmental Supportiveness (TOES).  Bundy (1999) | Children with developmental disabilities were less playful than their peers without developmental disabilities, regardless of the presence of siblings or gender  The ToP is both reliable and valid and may provide clinicians and researchers with a practical means of measuring the construct of playfulness | Reliability and validity of the TOES as a measurement of the environmental support of play was also supported  Data from 100% of the participants and raters conformed to the Rasch model  All items (100%) fit the model supporting that the ToP reflects a unidimensional construct of playfulness |
| Angelin et al. (2018) | -To compare the play performance  of preschool children in TD and children with CP, and  investigate the relationship between the level of func  tional mobility and manual function on play in these  children. | Cross sectional study | 96.2% | **Primary outcome measure:**  The revised Knox Preschool Play Scale (KPPSr) (Knox, 2002).  **Secondary Outcome Measure**  Gross Motor Function Classification System – Expanded and Revised (GMFCS – E&R) (Palisano, Rosenbaum, Bartlett, &  Livingston, 2008)  Manual Ability Classification  System (MACS), respectively (Eliasson et al., 2006). | The mean (15.20, SD 21.142) obtained by the study group was always lower than the mean (40.97 SD 8.977) obtained by the control group, presenting difference statistically evident between the groups in all dimensions, using a t-test for independent samples.  No significant associations between the manual function and functional mobility and the pretense/symbolic play and participation dimensions of the KPPSr. This suggests that these dimensions are not directly influenced by motor skills  in children with CP. | These results indicate that a greater impairment of manual function and functional mobility  led to poor engagement with recreational activities requiring those functions. |
| Wilkes-Gillan et al. (2016) | -To investigate if children and parents who had engaged in the parent-delivered intervention  18 months previously would benefit from an additional dose of technology-based intervention targeting  children’s social play skills. | Multiple case study | 96.2% | **Primary outcome measure:**  Test of Playfulness (Bundy, 2004)  **Secondary Outcome Measure**  Parent-rated Conners Comprehensive Behavior  Rating Scales (CCBRS)  (Conners, 2008). | Two children (pairs 2, and 5) showed an increase in their social play skills. The children with ADHD in these pairs had an eight- and 13-point difference in their pre- and post-intervention scores. Three children (pairs 1, 3 and 4) had a decrease in scores.  For the ToP scores of the playmates, three children (pairs 1, 2 and 3) showed consistent social play skills, one an increase in skill (pair 5) and one a decrease (pair 4). | described two main groupings of content through their interviews: (1) that as parents they felt better able to read and support their child and felt empowered as a result,  (2) that next steps for the intervention were necessary. |
| Barnes et al. (2017) | -To determine whether children with ADHD and their playmates maintained improvements in their social play skills, 12 months after their involvement in the RCT. The secondary aim was to evaluate the social skills and parent–child relationships of children with ADHD and their playmates. | Experimental study | 95.5% | **Primary outcome measure:**  Test of Playfulness (ToP)  (Bundy, 2004)  **Secondary Outcome Measure**  Social Skills Improvement System (SSIS Gresham & Elliott, 2008)  Parenting Relationship Questionnaire (PRQ) (Kamphaus & Reynolds, 2006)  Conners Comprehensive Behavior Rating Scales  (CCBRS)(Conners, 2008) | No significant change was shown between mean ToP scores of the children with ADHD in the home environment (time points A and C; t=0.96, P=0.36, d=0.68).  A significant decrease was shown between mean ToP scores of the children with ADHD in the clinic environment (time points B and D = -2.38, P=0.04, d= -0.57).  No significant difference was shown between mean ToP scores of the playmate group in the home (time points A and C; t = 0.25, P = 0.81, d = 0.48) or clinic (time points B and D; t = -1.63, P = 0.14, d = -0.74). | both groups scored within a developmentally appropriate range on all SSIS scales, with the ADHD group scoring significantly lower than the playmate group.  Children with ADHD scored within the average range for five of the seven PRQ scales. Playmates scored  within the average range for six of the seven PRQ scales. |

*Notes*. M - Mean: SD - Standard deviation; F = F ratio of ANOVA, *p* = probability value; ᶇᶈ² - partial eta square: effect size for ANOVA; g = Hedges’
